# Paradoxical melanoma in situ arising in a depigmented patch secondary to immunotherapy

**DOI:** 10.1016/j.jdcr.2026.05.020

**Published:** 2026-05-15

**Authors:** Karishma S. Shah, Yuna Kang, Caroline Opene

**Affiliations:** a Department of Dermatology, University of California, Irvine, Irvine, California; b Department of Pathology & Lab Medicine, University of California, Los Angeles, Los Angeles, California; c Division of Dermatology, Department of Medicine, University of California, Los Angeles, Los Angeles, California

**Keywords:** immunotherapy, melanoma associated vitiligo, melanoma in situ, PD-1 inhibitor therapy, pembrolizumab, vitiligo, vitiligo-like depigmentation

## Introduction

Vitiligo is an acquired pigmentary disorder and is the most common cause of depigmentation worldwide. The relationship between vitiligo and melanoma is complex, and it is important to understand which one was present first. There have been conflicting reports in the literature about the association between primary spontaneous vitiligo and overall melanoma risk. While genetic evidence suggests a lower risk for melanoma in patients with existing vitiligo, epidemiological evidence is inconsistent.^1^

Depigmentation occurring in patients who already have melanoma has been termed “melanoma-associated vitiligo (MAV).” The prevalence of MAV ranges in reports from about 2.8% to 16% and has been associated with a higher survival rate, even in patients with advanced disease.^2^ More recently, antimelanoma therapies, such as checkpoint inhibitors (CPIs), have been shown to induce vitiligo-like depigmentation (VLD). A recent study in patients with melanoma receiving anti-PD1 therapy found that those who developed VLD showed much more heterogeneity in their proteomic profiles when compared to patients who did not develop VLD.^3^ This may impact therapy effectiveness as current data suggests that the occurrence of VLD secondary to CPI therapy is associated with better treatment outcomes.^4^^,^^5^

## Case

**Fig 1:**
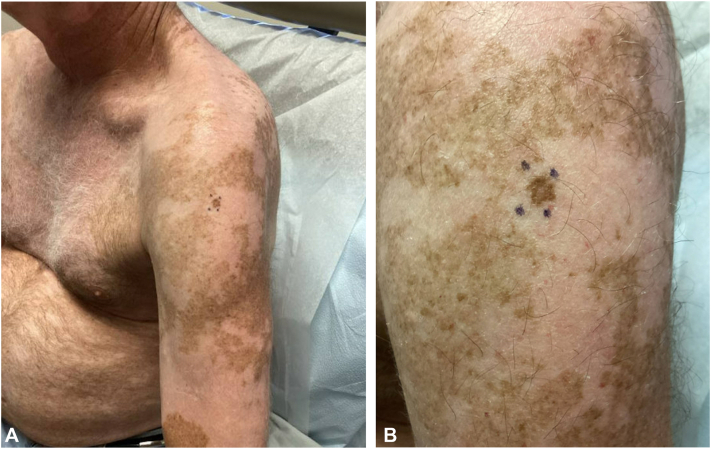
Clinical images from visit in August 2024 depicting a 1 cm irregularly pigmented brown macule on the left upper arm in a depigmented patch **(A, B)**.

**Fig 2:**
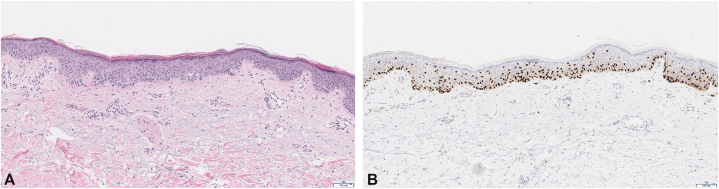
All of the histological images are at 10× power. Melanoma *in situ*, characterized by an atypical junctional/lentiginous melanocytic proliferation with Pagetoid upward scatter on hematoxylin and eosin **(A)** and SOX10 **(B)** immunohistochemical stain.

A 67-year-old man was referred to our dermatology clinic in February 2022 by his oncologist for total body skin examination. He was undergoing treatment with pembrolizumab and vibostolimab for metastatic stage IV melanoma with metastases to the pelvis, external iliac lymph nodes, and common femoral lymph nodes with as yet unknown primary. On initial examination, no primary cutaneous lesion was found. His subsequent course was complicated by severe psoriasiform drug eruption that cleared after stopping immunotherapy in December 2022. At that time, he had achieved remission as evidenced by stable positron emission tomography scan with normal inguinal nodes. In August 2023, the patient returned with widespread depigmented patches involving his trunk, arms, and legs that was concerning for VLD secondary to pembrolizumab. On a routine visit for skin check in August 2024, an irregularly pigmented 1 cm brown macule was noted on the left upper arm in a depigmented patch (Fig 1). Shave biopsy revealed melanoma in situ, a diagnosis supported by positive SOX10, tyrosinase, and PRAME immunohistochemistry stains (Fig 2). He underwent wide local excision with clear margins. He was most recently seen in clinic in February 2026 with no evidence of recurrence.

## Discussion

VLD has been reported in 11% of melanoma patients treated with pembrolizumab. Although there have been numerous studies looking at MAV in the absence of immunotherapy as a prognosticating factor, the studies evaluating the VLD secondary to immunotherapy are limited. To our knowledge, there is one study prospectively evaluating the appearance of vitiligo in patients receiving pembrolizumab. This study included 67 melanoma patients who were being treated with pembrolizumab, of which 17 developed vitiligo. The time to onset of vitiligo ranged from 52-453 days after the initiation of treatment, with a median of 7 infusions administered before onset. The difference in objective (complete or partial) tumor response between the vitiligo and nonvitiligo groups was significant with 71% of vitiligo patients achieving objective response compared to 28% of nonvitiligo patients.^4^ Notably, a retrospective study evaluated 5737 patients with melanoma being treated with a variety of immunotherapies, including pembrolizumab. The overall incidence of vitiligo was 3.4% and its development was associated with a significant increase in both progression free and overall survival.^5^ Similarly to these studies, our patient has been in remission from his original metastatic melanoma for over 2 years.

Although there is still no consensus on the mechanism underlying VLD secondary to CPI therapy, one theory has been proposed. It is thought that under normal circumstances, PD-1 mediates tolerance to melanosomal antigens and inhibits the formation of autoantibodies. However, in melanoma there is overexpression of melanocytic antigens in tumor cells. This, combined with PD-1 inhibition, leads to development of autoantibodies, causing autoimmune destruction of melanocytes and ultimately resulting in vitiligo. This theory is supported by studies, which show the infiltration of same clonal population of CD8 T cells in both tumor and vitiligo lesions. The circulation of antibodies against melanoma associated antigens has also been shown to be present.^6^ Additionally, this subset of circulating antibodies and memory T-cells against shared melanoma and melanocyte antigens may explain why VLD secondary to immunotherapy is a favorable prognostic indicator.

There are only a few cases in the literature that describe melanoma arising in a depigmented area. One report discusses 2 patients with a solitary depigmented patch that eventually developed melanoma. The authors hypothesized that this was due to the immune system recognizing melanoma antigens and attempting to destroy the diseased melanocytes.^7^ The other report describes a patient with longstanding primary spontaneous vitiligo who developed a melanoma within one of the vitiligo patches.^8^

Taken together, our case may represent a paradox—a patient whose delayed vitiligo-like depigmentation signaled the successful treatment of metastatic melanoma, yet who subsequently developed a new primary melanoma in situ in an area where melanocytes have been destroyed. This suggests that the destruction of melanocytes in VLD secondary to immunotherapy is incomplete and that there are residual melanocytes with enough oncologic potential to proliferate. Although our patient developed a durable response with pembrolizumab for his stage IV melanoma, which supports the current literature, he developed a new primary lesion.

Additionally, this case highlights the importance of collaboration between dermatologists and oncologists to provide comprehensive care for patients throughout their immunotherapy course. Where resources permit, we recommend patients on immunotherapy establish care with a local dermatologist to monitor and treat common CPI-associated cutaneous side effects. As we know, many of these side effects can be managed while the patient continues their life-saving medication.

## Conclusion

This case highlights the need to maintain a high level of suspicion for melanocytic atypia even in a depigmented area, especially in a patient with a past history of melanoma. There is a need for more studies to further investigate the impact of VLD secondary to immunotherapy on the development of atypical melanocytic proliferations. Lastly, dermatologists remain an integral part of the care team for patients on immunotherapy.

## Conflicts of interest

None disclosed.
